# Three-Dimensional Reflectance Traction Microscopy

**DOI:** 10.1371/journal.pone.0156797

**Published:** 2016-06-15

**Authors:** Jihan Kim, Christopher A. R. Jones, Nicholas Scott Groves, Bo Sun

**Affiliations:** Department of Physics, Oregon State University, Corvallis, Oregon, United States of America; Technion - Israel Institute of Technology, ISRAEL

## Abstract

Cells in three-dimensional (3D) environments exhibit very different biochemical and biophysical phenotypes compared to the behavior of cells in two-dimensional (2D) environments. As an important biomechanical measurement, 2D traction force microscopy can not be directly extended into 3D cases. In order to quantitatively characterize the contraction field, we have developed 3D reflectance traction microscopy which combines confocal reflection imaging and partial volume correlation postprocessing. We have measured the deformation field of collagen gel under controlled mechanical stress. We have also characterized the deformation field generated by invasive breast cancer cells of different morphologies in 3D collagen matrix. In contrast to employ dispersed tracing particles or fluorescently-tagged matrix proteins, our methods provide a label-free, computationally effective strategy to study the cell mechanics in native 3D extracellular matrix.

## Introduction

Cellular traction force plays an important role in many living systems, from wound healing [1, 2], immune response [3, 4] and cancer invasion [5] in animals, to the motility of simple organisms such as Dictyostelium discoideum [6, 7]. Cellular traction force is typically generated by actomyosin contraction, and then transmitted to the extracellular matrix (ECM) through cell-substrate adhesion sites [8]. This mechanical interaction between cells and ECM is not only crucial for cell migration [9], but also supports a two-way feedback which allows the cells to sense the rigidity of their local environment [5, 10, 11]. Since the pioneering work using wrinkling elastic substrate [12], many efforts have been devoted to measuring the contractility of cells as an important biophysical characterization. Most studies to date have been focused on cells plated on 2D surfaces. There are basically two different approaches for 2D traction microscopy. In the first approach, cells are cultured on soft substrates, such as polyacrylamide gels [13]. The substrates are coated or embedded with markers (such as fluorescent tracing beads), and the substrate deformation is measured by particle image velocimetry (PIV) or particle tracking velocimetry (PTV). In the second approach, cells are on top of arrays of bendable micropillars, and the bending angle of the pillars can be used to directly calculate the force exerted [14–16]. In more recent studies, it has been shown that even when the cell and substrate interface is 2D, traction field has a normal component and is truly 3D in nature [7, 17, 18].

Despite the success of 2D traction microscopy, we now realize that cells in 3D environment may exhibit very different biochemical and biophysical phenotypes, and 3D traction force microscopy is required to understand many physiological processes [19]. To measure cell contractility in 3D, micropillar arrays are no longer applicable, and most studies therefore employ tracing beads dispersed in the ECM as a natural extension of 2D traction microscopy [20–22]. However, this method also has potential limitations. First, tracing beads do not reveal the actual matrix remodeling induced by the cells [23], but rather interpolate the full deformation field from the nodes defined by the locations of the beads. Second, the spatial resolution of the measured deformation field is determined by the volume fraction of the tracing beads. In practice, one has to balance between the need for high particle density to achieve better resolution, and the need for lower particle density for accuracy in multi-particle tracking [22]. Finally, the biological effects of tracing beads must be considered. Beads that are physically absorbed in the ECM can be detached from the matrix by the cells and engulfed through endocytosis, causing error in the traction measurement [24]. When using beads that are permanently bound to the ECM, one has to consider the potential effects due to the particle surface chemical treatment. Rescently, another strategy explored in [23] takes advantage of fluorescently labeled fibrin gels to avoid using tracing beads. However, this method requires specially constructed proteins for each type of ECM, and it is unclear if the native ECM microstructure would be affected. Given these limitations, we are motivated to develop a label-free method to measure the 3D traction fields of mammalian cells using standard laser scanning confocal microscopes.

Our method is based on confocal reflection imaging of type I collagen-based ECM. Type I collagen is the dominant element of connective tissue. Reconstituted collagen gel has gained popularity as arguably the most employed *in vitro* model of 3D ECM [25], providing insights about morphogenisis [26], wound repair [27], and in particular, the mechanosensitivity of tumor growth and migration [5, 28]. The fibrous structure of collagen gel can be visualized by confocal reflection microscopy (CRM, [29, 30]), which eliminates the need for extra probes. Confocal reflection microscopy is highly polarized: fibers that extend in the vertical direction will be missed due to a small reflective cross-section [31, 32], and fibers that can be visualized have an extended point spread function along the optical axis. Taking advantage of these imaging features, we have developed a new strategy to measure the cell-induced matrix deformation, 3D reflectance traction microscopy, which is based on partial volume correlation (PVC) analysis of confocal reflection images. Our approach allows us to measure generic 3D deformation field from confocal reflection images, and presents a label-free strategy to quantify the cell contractility in truly 3D, tissue-mimicking configurations.

## Materials and Methods

### Cell culture and treatment

Rat tail collagen (Corning, Newyork, U.S.) is diluted with DMEM growth medium, phosphate-buffered saline (PBS, 10X), and sodium hydroxide (NaOH, 0.1M) to concentration of 2.0 mg/mL with pH 7.4. Low concentration of human breast carcinoma cells (MDA-MB-231) are mixed with 250 *μ*L of collagen solution and added to glass bottom dish (MatTek, Messachusetts, U.S.). The dish is kept in an incubator at 37°C with 5% CO_2_ level for two days. To release cellular traction force, we dilute cytochalasin-D (Sigma-Aldrich, Missouri, U.S.) with PBS to a 1:1000 ratio and add 3 mL of the solution to the sample.

### Confocal reflection microscopy

We use a 20X, 0.7 numerical aperture (NA), oil-immersion objective on a motorized inverted confocal microscope (DMI 4000B, Lecia, Germany). Two wavelengths of lasers are used to capture reflection images of collagen fibers (532 nm) and fluorescently labeled cancer cell cytoplasm (488 nm, CellTraker Green CMFDA Dye, Thermo Fisher Scientific). 2D confocal images (1024 × 1024 pixels or 366.6 × 366.6 *μ*m) are recorded in 0.5 *μ*m increments along the z-direction for a total of 90 to 120 *μ*m. To obtain deformation of fibers, image stacks are recorded before and after cytochalasin-D treatment. The time interval between these two image sets is 1.5 hours.

### Reconstruction of cancer cell membrane

To reconstruct the surface of cancer cells from confocal image stacks, we first perform deconvolution using Tikhonov-Miller method (DeconvolutionLab, Biomedical imaging group, Switzerland) and a point spread function (PSF) which is measured experimentally. The PSF is obtained by imaging single fluorescent beads (220 nm in diameter) embedded in cell-free collagen gels at the same excitation/detection wavelengths as the cell imaging. We then threshold the deconvolved cell images and the cell membrane is determined through isosurface interpolation (Matlab, MathWorks, U.S.).

### Strain field

We take numerical derivatives of the deformation field defined on a regular lattice to compute the strain tensor field:

$$\begin{array}{c} \varepsilon =(\begin{array}{ccc} \frac{\partial D_{x}}{\partial x} & \frac{1}{2}(\frac{\partial D_{x}}{\partial y}+\frac{\partial D_{y}}{\partial x}) & \frac{1}{2}(\frac{\partial D_{x}}{\partial z}+\frac{\partial D_{z}}{\partial x})\\ \frac{1}{2}(\frac{\partial D_{y}}{\partial x}+\frac{\partial D_{x}}{\partial y}) & \frac{\partial D_{y}}{\partial y} & \frac{1}{2}(\frac{\partial D_{y}}{\partial z}+\frac{\partial D_{z}}{\partial x})\\ \frac{1}{2}(\frac{\partial D_{z}}{\partial x}+\frac{\partial D_{x}}{\partial z}) & \frac{1}{2}(\frac{\partial D_{z}}{\partial y}+\frac{\partial D_{y}}{\partial z}) & \frac{\partial D_{z}}{\partial z} \end{array}) \end{array}$$

where *D*_*x*_, *D*_*y*_, and *D*_*z*_ represent deformation field matrix along x, y, and z-direction. The strain magnitude then can be calculated by

$$\begin{array}{c} \varepsilon =\sqrt{\varepsilon :\varepsilon }=\sqrt{\varepsilon_{ij}\varepsilon_{ij}} \end{array}$$

### Normal and tangential components of deformation field

To quantify the relation between deformation and distance from the cell membrane, we calculate the normal distance from each point in space to the cell membrane. First, we define a discretized binary field corresponding to the cell membrane in the same grid where deformation field is defined. This is done by looking for boundary points separating the cells (values equal to 1, as obtained from the deconvolved and thresholded fluorescent images) and the background (values equal to 0). The normal distance and the normal and tangential components of the deformation fields are then calculated as described in the main text.

## Results

**Fig 1:**
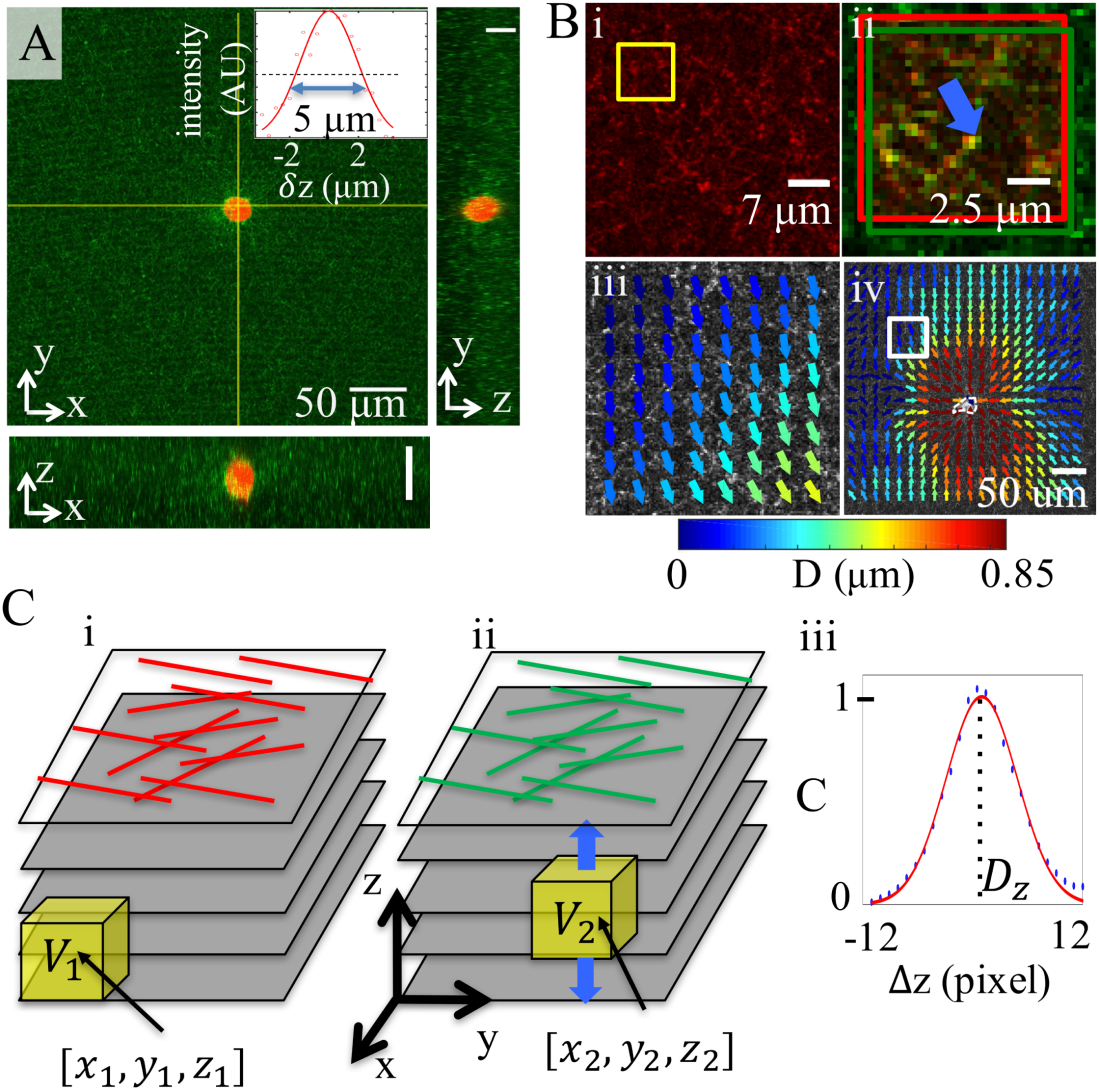
The work flow of three-dimensional reflectance traction microscopy. (A) A 2D cross-section from multichannel confocal imaging of a breast cancer cell (MDA-MB-231, red) embedded in 3D collagen matrix (green). Inset, the intensity profile of a typical fiber along the z-direction (symbols) and its Gaussian fit (solid curve). The full width half maximum is 5.3 *μ*m. (B-C) Schematic diagram of partial volume correlation (PVC) algorithm to measure 3D deformation before and after the cells are treated with cytochalsin-D. (B) PVC starts with 2D particle image velocimetry (PIV) analysis. (i) A portion of a confocal reflectance slice showing collagen fibers before cytochalsin-D treatment. The yellow square encloses a sub-window image to be compared with its counterpart after cytochalsin-D treatment. (ii) FFT based 2D image correlation calculates the average displacement of the sub-window before (red) and after (green) cytochalsin-D treatment. (iii) The 2D deformation field of the region in (i) on a spatial grid of 16 pixels, or 5.7 *μ*m. The arrows are color coded by the magnitude of the deformation field. (iv) The 2D deformation field of the full confocal reflectance slice. The white square corresponds to the region in (i) and (iii). White dashed line represents the cell membrane. The arrows are color coded by the magnitude of the deformation field. (C) Calculation of 3D deformation following 2D PIV described in (B). (i) Confocal reflectance image stacks of traction stressed collagen matrix (before cytochalsin-D treatment). The yellow box encloses a sub-volume *V*_1_ (32 × 32 × 20 pixels or 11.4 × 11.4 × 10 *μ*m, centered at [*x*_1_, *y*_1_, *z*_1_]), which is to be compared with its counterpart *V*_2_ after cytochalsin-D treatment. (ii) The sub-volume *V*_2_ has the same size as *V*_1_, and is centered at [*x*_2_, *y*_2_, *z*_2_] = [*x*_1_ + *D*_*x*_, *y*_1_ + *D*_*y*_, *z*_1_ + Δ*z*]. Here Δ*z* varies between -12 to 12 pixels. *D*_*x*_ and *D*_*y*_ are 2D deformation field (calculated in (B)) averaged over *V*_1_. (iii) Normalized volume correlation *C*(Δ*z*) between *V*_1_ and *V*_2_ as a function of Δ*z* (blue dots). 5 points near the maximum of *C*(Δ*z*) is fitted with a Gaussian function (red line). The center of the Gaussian function determines *D*_*z*_, which represents the average deformation in the z-direction of the sub-volume *V*_1_.

Partial volume correlation (PVC) algorithm offers a computationally inexpensive alternative to direct volume correlation (DVC, [33, 34]) to analyze the 3D deformation fields from confocal reflection images. We first illustrate the work flow of PVC in Fig 1 with an example data set. Fig 1A shows a typical 2D cross-section from a multichannel confocal reflection image of a breast cancer cell (MDA-MB-231) embedded in 3D collagen matrix. Fluorescent-labeled cell cytoplasm is shown in red and confocal reflection image of collagen fibers is shown in green. The inset shows the intensity profile of a typical fiber along the z-direction, where the full width half maximum (FWHM) intensity is about 5 *μ*m. Note that this is much larger than the typical deformation induced by the cells (≤ 2 *μ*m, see for example S1 Fig). 2D slices are taken both before and after we treat the cell with cytochalasin-D which disrupts the formation of actin microfilaments and causes a cell to release traction forces. PVC algorithm consists of two major steps: 2D lateral analysis and z-directional analysis. In the first step, we apply Fast Fourier Transformation (FFT, [35]) or Direct cross-correlation (DCC, [36]) based particle image velocimetry (PIV) analysis to obtain the 2D deformation field [37, 38]. As demonstrated in Fig 1B, two images chosen from each stack (before and after cytochalasin-D treatment) at the same height are compared through continuous sub-window deformation. Fig 1B(i) shows a portion of a confocal reflectance slice of collagen fibers before cytochalasin-D treatment. The yellow square indicates a sub-window of Fig 1B(i) (32 × 32 pixels or 11.4 × 11.4 *μ*m). In Fig 1B(ii), this sub-window (red) is compared with the image slice after cytochalsin-D treatment (green). FFT or DCC based algorithm calculates the average displacement of the sub-window which is shown as the color coded arrow. We repeat the sub-window analysis for the entire slice by continuously stepping sub-windows which gives a coarse-grained 2D deformation field [37]. For the example here, the sub-window moves at a step of 16 pixels, and sets the spatial resolution of displacement field to be 16 pixels or 5.7 *μ*m. Due to the porous nature of collagen matrix, we find that iterative refining of sub-windows is generally not necessary.

Fig 1B(iii) shows the deformation field of the region in Fig 1B(i). The deformation field of the full field of view from typical confocal imaging is shown in Fig 1B(iv). White square corresponds the same region in Fig 1B(i) and 1B(iii). The arrows are color coded by the magnitude of the local deformation field and cancer cell membrane is outlined with white dashed line at the center. The 2D PIV analysis is applied to the entire stack at each height along the z-axis.

With the 2D deformation field known for each z-position, we now move on to calculate the 3D deformation field. Similar to the sub-window analysis described above, we first take a sub-volume *V*_1_ from the image stack before cytochalsin-D treatment. *V*_1_ is centered at [*x*_1_, *y*_1_, *z*_1_], and has a size of [*l*_*x*_, *l*_*y*_, *l*_*z*_]. It is natural to set *l*_*x*_ and *l*_*y*_ to be the same size as the 2D sub-windows, so we choose *V*_1_ to be 32 × 32 × 20 pixels or 11.4 × 11.4 × 10 *μ*m. *V*_1_ is compared with *V*_2_, a sub-volume taken from image stacks after cytochalasin-D treatment. *V*_2_ is centered at [*x*_2_, *y*_2_, *z*_2_] = [*x*_1_ + *D*_*x*_, *y*_1_ + *D*_*y*_, *z*_1_ + Δ*z*] where *D*_*x*_ and *D*_*y*_ are average 2D deformation vectors within *V*_1_ obtained from PIV analysis (Fig 1B). The center position of *V*_2_ is varied by Δ*z* which goes from −*M*_*z*_ to *M*_*z*_, where *M*_*z*_ is set by the largest possible deformation in the z-direction. We choose *M*_*z*_ to be 12 pixels or 6 *μ*m (Fig 1C(ii)). To compare *V*_1_ and *V*_2_, we compute normalized volume correlation *C* as a function of Δ*z*. Notice that *V*_1_ and *V*_2_ are 3D matrices with matrix indexes [*i*, *j*, *k*]. *C* is defined as

$$\begin{array}{c} C=\frac{\left(V_{1}-<V_{1}>\right)*\left(V_{2}-<V_{2}>\right)}{\sqrt{\left(V_{1}-<V_{1}>\right)*\left(V_{1}-<V_{1}>\right)}\sqrt{\left(V_{2}-<V_{2}>\right)*\left(V_{2}-<V_{2}>\right)}} \end{array}$$ (1)

where the operation ‘*’ is defined as

$$\begin{array}{c} X*Y=\sum_{\left[i,j,k\right]}X_{i,j,k}Y_{i,j,k} \end{array}$$ (2)

In practice *C*(Δ*z*) can be easily obtained from numeric matrix convolutions. A typical result of *C*(Δ*z*) is plotted in Fig 1C(iii) (blue dots). The maximum of the function happens when Δ*z* = *D*_*z*_, where *V*_1_ and *V*_2_ are most similar. *D*_*z*_ corresponds to the coarse-grained deformation in the z-direction. To better locate the peak position of *C*(Δ*z*), we fit the curve with a Gaussian function around the maximum (Fig 1C(iii), red lines), and define the z-deformation *D*_*z*_ as the center of the Gaussian function. This additional step has been shown to improve the accuracy to sub-pixel resolution [37, 39].

By applying the partial volume correlation algorithm to the entire image stack, we can finally obtain the full 3D deformation field **D**(**r**) at sub-pixel accuracy and a spatial resolution set by the size of the sub-volume. Note that for typical experimental data, we often need to perform a spatial low-pass filter on the output of PVC analysis to remove outliers due to image imperfection. In the experimental examples shown below, we have employed discretized spline smoothing [40] with smoothing parameters automatically determined [41].

**Fig 2:**
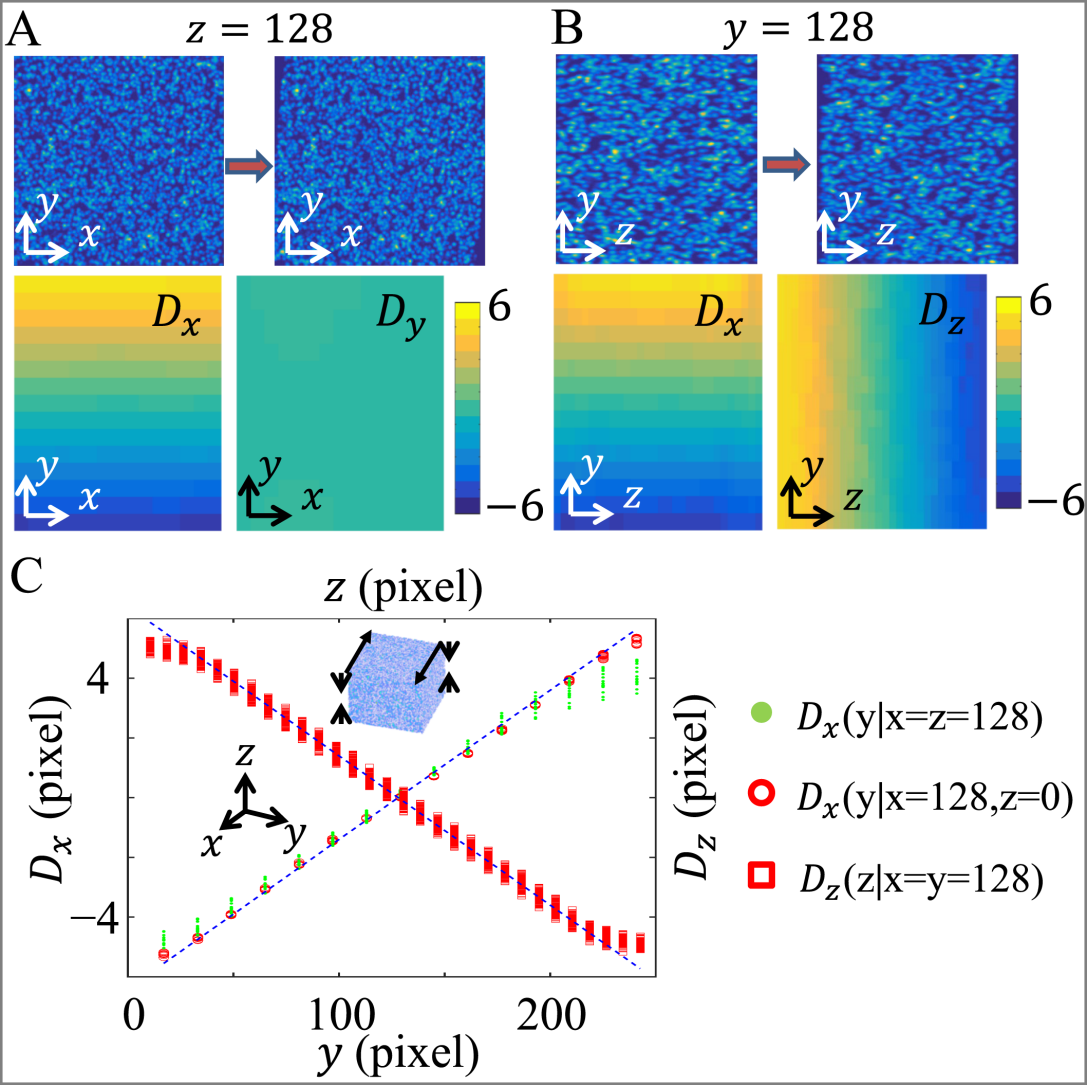
Partial volume correlation (PVC) algorithm applied to virtual image stacks. An initial virtual image stack contains 100,000 particles which are randomly located in a cube of 256 × 256 × 256 pixels. The cube is then subject to uniform compression along the z-direction and shear along the x-direction, leading to a deformed virtual image stack. (A) Deformation in the xy-plane. Top panels show the virtual image slice before and after applying deformation. Bottom panels show the projection of PVC calculated 3D deformation fields. (B) Deformation in the yz-plane. Top panels show the virtual image slice before and after applying deformation. Bottom panels show the projection of PVC calculated 3D deformation fields. (C) Comparison of PVC calculated 3D deformation fields **D**(**r**) (open symbols) with accurate values (blue dashed lines). In all cases tested, the deviation from accurate values is less than 5%.

In order to test the performance of the PVC method, we first apply the PVC algorithm to computer generated artificial data sets. To generate a virtual image stack, 100,000 particles are randomly dispersed in a cube of 256 × 256 × 256 pixels with each particle generating a 3D Gaussian point spread function (Gaussian width equals to 2 pixels). The virtual stack is then obtained by direct summation of point spread functions from all particles. To generate a deformed virtual image stack, we move the particle centers based on prescribed deformation field and recalculate a second image stack by summing over all particle intensity profiles. For this test, we apply a uniform compression along the z-direction and shear along x-direction. Fig 2A shows the 2D slices at *z* = 128 before and after deformation and the result of PVC analysis is shown in the lower panels. Similarly, Fig 2B shows the 2D slices and PVC result at *y* = 128. In both cases, all components of the deformation field follow the expected spatial profiles, and errors are most pronounced with large deformations in the z-direction. To better illustrate the comparison between numerical calculations and the prescribed deformation field, we plot the PVC results along three lines. As shown in Fig 2C, PVC results agree with the accurate values within a 5% error. The error is systematic, with large deformation fields being underestimated. However, the sub-pixel error is typically much smaller than experimental uncertainties. The error estimates also agree with tests using real confocal reflection images, as summarized in the supplementary information (S1 and S2 Figs).

**Fig 3:**
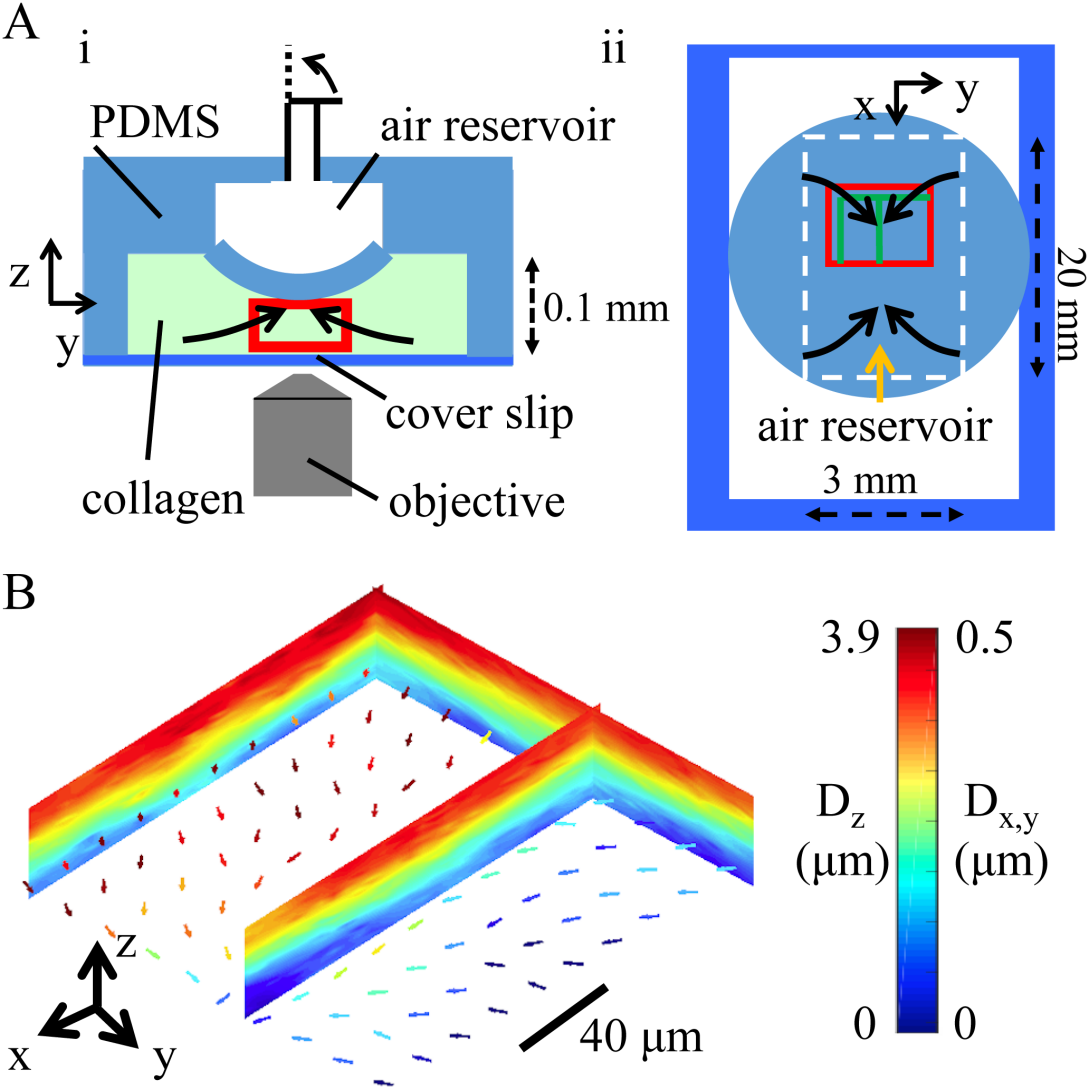
Three-dimensional reflectance traction microscopy applied to a mechanically deformed collagen matrix. (A) Schematic illustration of the experimental setup with side view (i) and top view (ii). A polydimethylsiloxane (PDMS) channel (20 mm × 3 mm) is in contact with collagen gel and is inflated with air. When air is released, the squeezed collagen gel relaxes to a stress-free state, resulting in a deformation field which is expected to follow the arrows in (i) and (ii). The red rectangle encloses the volume of the confocal imaging field of view. (B) The 3D deformation field calculated with PVC algorithm. Deformation along z-axis (*D*_*z*_) of three selected cross-sections (corresponding to the green lines in A(ii)) are shown as color maps. The lateral deformation field (*D*_*x*_ and *D*_*y*_) of a selected horizontal cross-section is shown as arrows. The arrows are color coded by the magnitude of the lateral deformation field $D_{x,y}=\sqrt{D_{x}^{2}+D_{y}^{2}}$.

After testing PVC algorithm with virtual data, we then apply the method to genuine confocal reflection data sets of a collagen gel under controlled mechanical deformation. Fig 3A illustrates schematic design of the experiment. A chamber device is made with a polydimethylsiloxane (PDMS) cover bonded to a thin glass cover slip. All chamber surfaces are pretreated with tissue adhesive (Cell-Tak, Corning) to ensure tight binding with collagen matrix. We fill the chamber with neutralized collagen solution and let the gel form inside the chamber. The PDMS cover also includes an air reservoir with its bottom surface in contact with collagen matrix. The bottom surface of PDMS air reservoir can be deformed by injecting and removing air which is controlled by a syringe pump. First, a confocal reflection 3D stack is taken when the collagen gel is compressed by the air-inflated PDMS chamber. After gently deflating the chamber, we take a second confocal image stack when the collagen gel is no longer compressed. To register the largest deformation, we take confocal reflectance images near the center of the PDMS chamber. The field of view is indicated by the red boxes in Fig 3A(i) and 3A(ii), which are the side and top view of the experimental setup. From stressed to stress-free states, the expected deformation is shown as arrows in Fig 3A(i) and 3A(ii). The 3D deformation field calculated with PVC algorithm is shown in Fig 3B. The color map slices represent deformation field along z-direction (*D*_*z*_) on three x-z cross-sections (as indicated by green lines in Fig 3A(ii)). The lateral deformation in xy plane (*D*_*x*_, *D*_*y*_) is shown as color coded arrows and the color (blue to dark red) is proportional to the magnitude of lateral deformation field $D_{x,y}=\sqrt{D_{x}^{2}+D_{y}^{2}}$. The result shows that the calculated deformation field agrees with the expected pattern.

**Fig 4:**
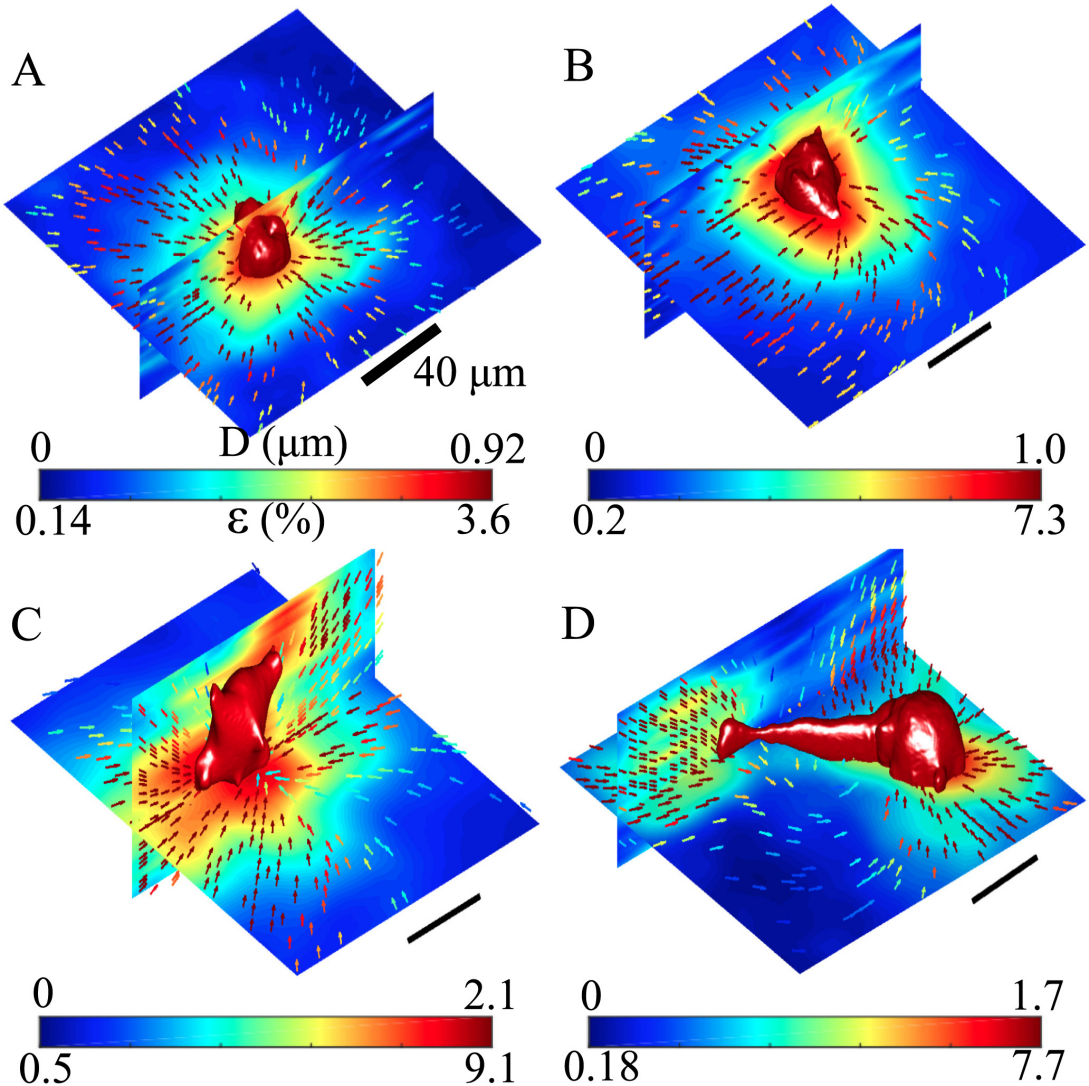
Three-dimensional reflectance traction microscopy reveals the traction field of breast cancer cells in 3D collagen matrix. (A-D) The strain magnitude *ε* and deformation field **D** measured around four isolated MDA-MB-231 cells before and after the cells are treated with cytochalsin-D. *ε* of orthogonal cross-sections are shown as color maps. **D** projected on to these cross-sections are shown as arrows. The cell surfaces (illuminated red surfaces) are reconstructed from isosurface rendering of fluorescently labeled cytoplasms. The arrows are color coded by the magnitude of the projected deformation fields. (A) and (B) demonstrate deformation and strain fields around cells with rounded morphology. (C) and (D) show the results around cells with larger aspect ratios.

Confocal reflection imaging combined with partial volume correlation method provides a label-free strategy to measure 3D traction force exerted by mammalian cells in tissue-mimicking environments. To demonstrate the applicability, we have studied the traction field of isolated human breast cancer cells (MDA-MB-231) embedded in collagen matrix by comparing the confocal reflection images before and after cytochalasin-D treatment. As shown in Fig 4, MDA-MB-231 cells exhibit a variety of morphologies after cultured in collagen gel for 24 hours. We have chosen to show two rounded (Fig 4A and 4B) and two elongated (Fig 4C and 4D) cells. For each cell, we have measured the deformation field **D**(**r**) (arrows, color coded by the magnitude of deformation *D*), and calculated the strain magnitude *ε*(**r**) (color maps, color coded by *ε*). It is evident that the traction fields are highly regulated by cell morphologies, which agrees qualitatively with previous measurements using tracing beads.

**Fig 5:**
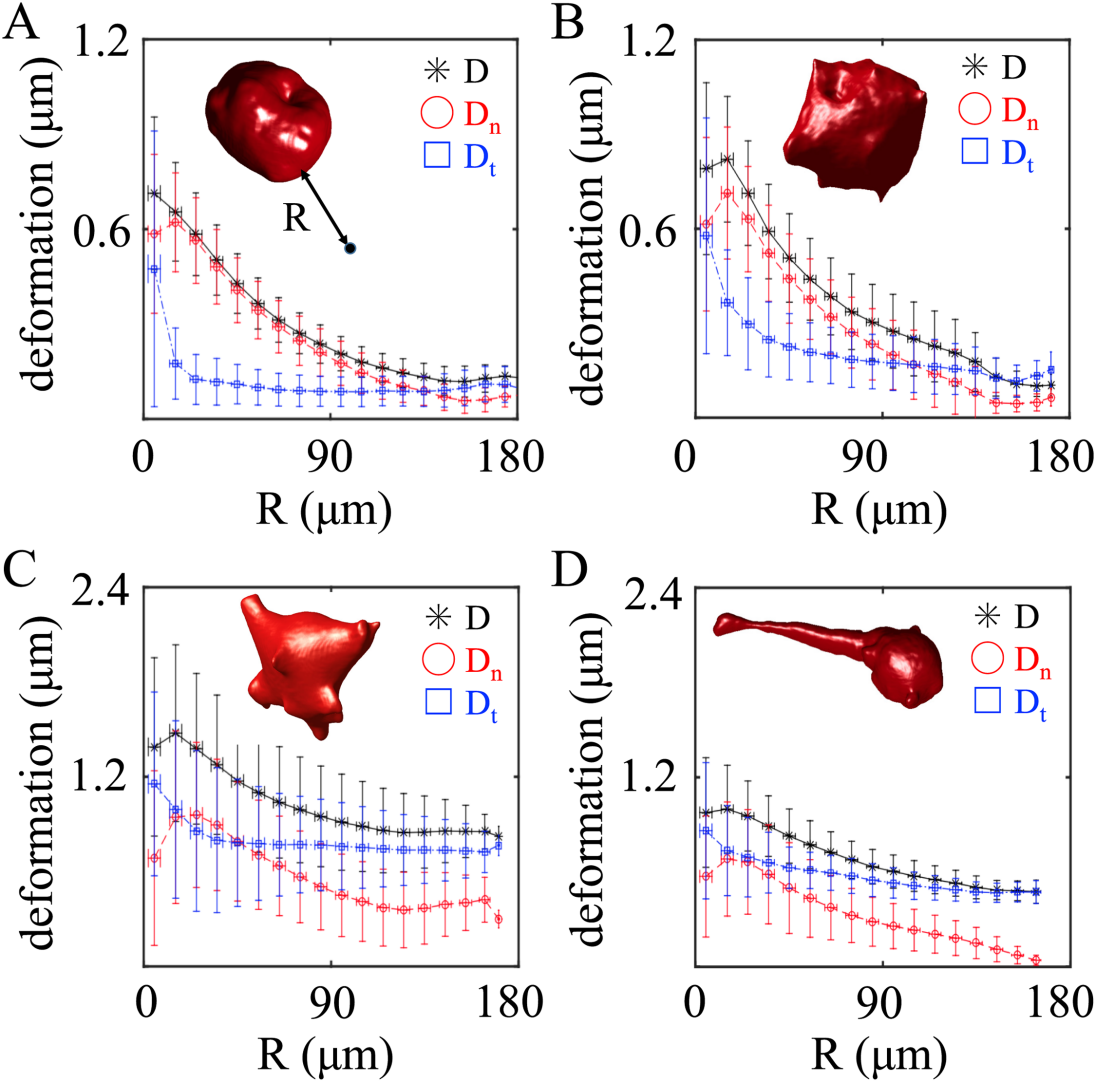
Quantifying the pattern of traction field by decomposing the deformation field into normal and tangential components. Plots showing the magnitude of the total deformation (*D*), normal (*D*_*n*_) and tangential (*D*_*t*_) components as a function of the distance to the cell membrane (*R*). A-D corresponds to the cells in Fig 4A and 4D respectively.

In order to further quantify the deformation field, we have decomposed the deformation field into normal and tangential components. Specifically, for each point **r**_*A*_ near the cell, we first locate the point **r**_*B*_ on the cell membrane which has the shortest euclidean distance to **r**_*A*_. We define *R* to be the length of Δ**r** = **r**_*B*_ − **r**_*A*_. For the normal component, we define $D_{n}=\frac{D\cdot \Delta r}{\left|\Delta r\right|}$, and for the tangential component, **D**_*t*_ = **D** − **D**_*n*_, where **D** is the deformation field at **r**_*A*_. To calculate *D*, *D*_*n*_ and *D*_*t*_ as a function of *R*, we have further binned the distance *R* with a width of 10 *μ*m. Error bars in Fig 5 show the average and standard deviation of *D*, *D*_*n*_ and *D*_*t*_ within each bin of the distance *R*. Fig 5A–5D correspond to the deformation fields of the same cells in Fig 5A–5D. Cells with rounded morphology generate smaller matrix deformation, and the normal component is dominant. On the other hand, cells of elongated morphology generate larger deformation, and the deformation is mostly in the tangential direction. This is consistent with previous reports that elongated fibroblast cells in 3D exert mostly shear rather than normal stress [20]. In addition, we have also computed the normal projection of the strain tensor. As shown in the supplementary material (S4 Fig), cells exert both pulling and pushing forces at the same time, suggesting that cytoskeleton contraction and membrane protrusion are tightly integrated local events.

Using linear approximations we have also estimated the cell surface traction stress **T** and cell contractility *C*, defined as total magnitude of cell contraction forces. The supplementary material shows the component of traction normal to the cell surface for several cells (S5 Fig) and illustrates the magnitudes of pushing and pulling tractions. The values for cell traction are consistent with recent results where tracing beads were used with cells embedded in 3D collagen gels [42]. The smaller contractility reported in S5 Fig could be due to the fact that linear elastic approximation is not an accurate representation of collagen gels.

## Discussion

We have presented a label-free approach to measure the 3D deformation field of mammalian cells in tissue-mimicking environments. Our image analysis, the partial volume correlation (PVC) method, is a hybrid of 2D and 3D image correlation based velocimetry. The method is computationally inexpensive, and offers sub-pixel accuracy when combined with confocal reflectance microscopy. The success of PVC analysis relies on best choice of parameters, and the more one knows about the general properties of the deformation field, the easier it is to find the optimal parameter sets [37]. In practice, the most important parameter is the sub-window and sub-volume size. They need to be large enough to contain enough features for image comparison, but also small enough to detect spatial variations of the deformation field on small length scales. The step size for continuously moving the sub-volume should be as small as possible given computational limits. However, due to the intrinsic coarse-graining by the sub-volume, and the smoothing in the post-processing, it is generally not necessary to set the step size smaller than half of the sub-volume size, or the Nyquist frequency.

The performance of PVC is limited by several factors. First, as we have shown in Fig 2, large deformation in the z-direction may cause underestimation of the lateral displacement. This is partially rectified by using confocal reflectance imaging, which has a very elongated point spread function along the z-direction [32]. Additional strategies include implementing iterative PVC: the z-displacement from previous iteration is used to offset the sub-windows for 2D PIV, and applying direct volume correlation to set the initial values. Second, PVC, at least in its current form, requires a relatively small strain field. Large strains (typically >15%), especially those involving 3D rotations, will cause inaccurate results. However, these limiting cases rarely appear in the traction fields of cells. We have shown that 3D reflectance traction microscopy characterize the contractility of breast cancer cell in type I collagen gel. For both types of cell morphologies, the magnitudes of deformation and strain fields are well below the limit of PVC.

With 3D reflectance traction microscopy, we have shown the ECM deformation field induced by MDA-MB-231 cells in native collagen matrix. The pattern of strain field is strongly correlated with cell morphology, which is consistent with previous reports [22]. Cells with rounded morphology generate weaker, and more spatially isotropic strain fields. Cells with elongated morphology generate stronger contraction which has a dipole pattern. At large distance *R* from the cell membrane, the average deformation and strain fields decay monotonically with *R*. However, significant fluctuations are observed which can be attributed to the structural and mechanical heterogeneity of collagen gel [30, 43], irregular cell shape [20], as well as the non-uniform distribution of cell-matrix adhesions [44].

As highlighted recently [23], directly visualizing the ECM fibers not only allows one to calculate the cell-induced deformation field, but also reveals details about the ECM microsctructural remodeling. Compared with the previously described methods [23], 3D reflectance traction microscopy provides such information without fluorescently labeling the ECM proteins, and only slightly increases the noise level. This label-free method does not require special preparation of the ECM, and may therefore have a wider range of applications, such as ECM with high protein concentrations and ECM from real tissue samples [45]. On the other hand, 3D reflectance traction microscopy is limited by the speckle-based imaging, particularly with thin fibers such as those in peptide gels. In these cases, tracing beads and fluorescently labeled matrix proteins are the preferred methods. As complimentary to existing strategies, more work is needed in the future to extend the applicability of 3D reflectance traction microscopy.

## Supporting Information

S1 Fig Systematic errors of reflectance traction microscopy. In order to estimate the noise due to systematic errors in the reflectance traction microscopy, we have taken 3 image stacks of a collagen gel sample without cells at 1 hour intervals. The image stacks have 1024 × 1024 × 60 pixels, where each voxel measures 0.36 × 0.36 × 0.5 *μ*m. Using the first stack (0 hour) as a reference, we have computed the 3D deformation field and strain tensor on a spatial grid of 16 × 16 × 8 at 1 hour and 2 hour time points. (A) The magnitude of 3D deformation field at the horizontal middle plane of the imaging volume at 2 hours. Inset: average deformation along each direction. Red: *D*_*x*_, Green: *D*_*y*_, Blue: *D*_*z*_. Overall, the magnitude of deformation due to systematic errors (such as mechanical drifts, temperature fluctuations) is less than 0.5 *μ*m over 2 hours. (B) The magnitude of strain magnitude at the horizontal middle plane of the imaging volume at 2 hours. Inset: average strain tensor along each principle directions. Red: *ε*_*xx*_, Green: *ε*_*yy*_, Blue: *ε*_*zz*_. Overall, the systematic errors of strain field is less than 1% over 2 hours.
(PDF)

S2 Fig Error of reflectance traction microscopy due to matrix remodeling. The ECM can be densified or degraded by cellular activities. To determine the effect of the ECM heterogeneity in the precision of reflectance traction microscopy, we have performed the test on simulated data sets. First of all, we have taken a section of confocal reflection images *I*_*raw*_ (128 × 128 × 64 pixels, available from https://github.com/bosunorst/Partial-Volume-Correlation/), and computationally translated *I*_*raw*_ by Δ*l* pixels in all directions. We then applied PVC to compute the (theoretically uniform) deformation field [*D*_*x*_, *D*_*y*_, *D*_*z*_] on a grid with 16 × 16 × 8 pixels spacing. Statistics of [*D*_*x*_, *D*_*y*_, *D*_*z*_] then estimate the error of reflectance traction microscopy. (A) The means and standard deviations of [*D*_*x*_, *D*_*y*_, *D*_*z*_] at varying Δ*l*. The errors of the deformation fields are within 5%. Inset: a 2D section of the image stack *I*_*raw*_. To simulate remodeled ECM, we have randomly chosen 110 cubes within *I*_*raw*_. These cubes each have a size of 8 × 8 × 8 pixels. In total these cubes occupy ≈4% volume of *I*_*raw*_. We fill the cubes by the value that corresponds to bright pixels (the upper 2% intensity in *I*_*raw*_) to simulate images of densified ECM *I*_*den*_, or we fill the cubes by the value that corresponds to dark pixels (the lower 2% intensity in *I*_*raw*_) to simulate images of degraded ECM *I*_*deg*_. Similar to (A), we have applied PVC on computationally translated *I*_*den*_ and *I*_*deg*_, and the results are shown in (B-C). (B) The means and standard deviations of [*D*_*x*_, *D*_*y*_, *D*_*z*_] at varying Δ*l* for densified ECM. The errors of the deformation fields are within 5%, except when Δ*l* > 4 pixels, where the errors are approaching 10%. Inset: a 2D section of *I*_*den*_. (C) The means and standard deviations of [*D*_*x*_, *D*_*y*_, *D*_*z*_] at varying Δ*l* for degraded ECM. The errors of the deformation fields are within 5%. Inset: a 2D section of *I*_*deg*_.
(PDF)

S3 Fig Comparison of 3D deformation field measured with different methods. We have compared reflectance traction microscopy side by side with two other popular approaches: particle tracking velocimetry (PTV) and direct volume correlation (DVC, [33]). (A) We have seeded low density fluorescent tracing particles (red, 0.2 *μ*m diameter, highlighted by the arrows) in collagen gels (green) containing MDA-MB-231 cells (red). The particle density is kept low so that (1) it does not bias the confocal reflection image; (2) it avoids ambiguities in trajectory reconstruction. Scale bar: 100 *μ*m. (B) We have calculated deformation field induced by MDA-MB-231 cells on a spatial grid of 4 × 4 × 4 pixels, with each voxel measures 0.36 × 0.36 × 0.5 *μ*m. We have applied both PVC and DVC methods on the reflectance images, and (linearly) interpolated the deformation field at particle locations to obtain **D**^*PVC*^ and **D**^*DVC*^. On the other hand, we have calculated the particle displacements using their fluorescent images directly with PTV, resulting in **D**^*PTV*^. Results of ≈ 100 particles from three experiments are shown in B, where we compare **D**^*PVC*^, **D**^*DVC*^ with respect to **D**^*PTV*^. The mean square deviations between PVC and PTV are 0.11 *μ*m in x-direction, 0.08 *μ*m in y-direction, 0.51 *μ*m in z-direction. The mean square deviations between DVC and PTV are 0.11 *μ*m in x-direction, 0.1 *μ*m in y-direction, 0.47 *μ*m in z-direction. Therefore PVC and DVC give very close results, and their deviation from PTV is comparable to the noise ground set by systematic errors (S1 Fig).
(PDF)

S4 Fig The PDMS device designed to create programable deformation of collagen gel. (A-B) The top and side view of the device. The red dashed lines indicate air channel and the blue lines show the region where collagen gel is placed. There is a thin PDMS membrane between air channel and collagen matrix. The membrane can be expanded or shrunk by controlling air volume through the tube. Scale bar: 10 mm. (C) Side view of the confocal reflection imaging of the sample. Green channel shows collagen fibers and red channel shows the PDMS membrane surface. This cross sectional image shows that the PDMS surface is slightly asymmetric due to the fabrication processes.
(PDF)

S5 Fig Structural and mechanical characterization of collagen gel. (A) The rheological modulus of the collagen gel as a function of frequency. Storage modulus *G*′ is plotted with solid circles and loss modulus *G*′′ is plotted with open circles. Measurements were taken using an AR 2000 rheometer with a 20 mm steel plate and Peltier plate for temperature control. Immediately after neutralization, collagen solution was injected between the plates, which had been preheated to 37°*C*. Silicone oil was allowed to polymerize for one hour before rheological measurements. Error bars show the standard deviation of five separate frequency sweeps on the same sample. (B) A sample confocal image slice with red circles representing pores in the collagen network. The pore size *d*_*p*_ is defined as the diameter of the largest circle that can be drawn to fit inside the pore. 240 pores were randomly selected and the circles were drawn manually. (C) The pore size distribution of the collagen gel. Mean pore size is 4.95 *μ*m. (D) Density fluctuation of the collagen gel as measured by 2-point intensity correlation *g*(*r*) [30]. Double exponential fit is given by *g*(*r*) = 0.8*exp*(−*r*/0.34*μ*m) + 0.2*exp*(−*r*/1.9*μ*m).
(PDF)

S6 Fig Normal projection of strain field. The traction field of cells can be qualitatively characterized by the normal projection of the strain field on to cell membrane. From the strain field *ε*_*ij*_, and the normal direction **n** of cell surface, we have calculated the normal projection *ε*_*nn*_ = **n** ⋅ *ε* ⋅ **n**. We have shown *ε*_*nn*_ of 8 cells on the same spatial and color scales (scale bar: 40 *μ*m). Cells exert both pulling (*ε*_*nn*_ < 0) and pushing (*ε*_*nn*_ > 0) forces, corresponding to different cellular activities, such as newly formed protrusion and active contraction.
(PDF)

S7 Fig Cell surface traction and cell contractility. We calculate the cell traction assuming that the collagen network is an isotropic, homogeneous material. We make the linear elastic approximation because strain magnitudes are small (<5%). Using these approximations, the Cauchy stress tensor *σ* is given by *σ*_*ij*_ = 2*Gε*_*ij*_ + λ*Trace*(*ε*)*δ*_*ij*_ where λ = 2*Gν*/(1 − 2*ν*), *G* is the shear modulus, *ν* is the Poission ratio, and *δ*_*ij*_ is the Kronecker delta [46, 47]. We take *G* = 50 Pa and *ν* = 0.2 which is consistent with previous experimental results [47–50]. The traction **T** at the cell surface is calculated from the stress tensor using the Caucy relation **T** = **n** ⋅ *σ* where **n** are the directions normal to the cell surface [46, 47]. We generate a finite element mesh to represent the cell surface and then calculate the surface normal and traction for each discrete face. The normal component of traction *T*_*n*_ = **T** ⋅ **n** is shown for nine cells on the same spatial and color scale (scale bars: 40 *μ*m). Negative normal traction represents pulling and positive normal traction represents pushing. We have found that the normal traction is generally the largest component of the total traction and that pulling tractions are generally stronger than pushing. Typical surface tractions in our experiments are ∼5–10 Pa which is consistent with previous results for cell traction in collagen gels [42], but much smaller than mean tractions ∼300 Pa reported for cells in synthetic PEG hydrogels [20]. In addition, we have calculated the contractility for each cell, which is defined as the total magnitude of force projected onto the direction of the center of mass of the cell *C* = ∑*A* **T** ⋅ **R**_*cm*_, where *A* is the area of each face, **R**_*cm*_ is the direction of the center of mass, and the sum is over all faces. Typical contractility for our experiments is ∼15 nN which is slighlty smaller than the previously reported value of ∼45nN for cells in 3D collagen gels [42]. The discrepancy could arise from linear elastic approximation used here, compared with the constitutive equation and regulation schemes used in [42].
(PDF)
