# Supplementary figures and images for: Three-Dimensional Reflectance Traction Microscopy
Source: PLoS One. 2016 Jun 15;11(6):e0156797. doi: 10.1371/journal.pone.0156797 (PMC4909212; doi:10.1371/journal.pone.0156797)

A

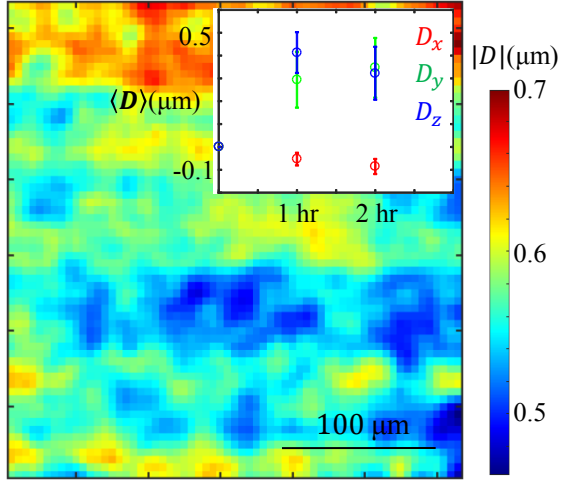

B

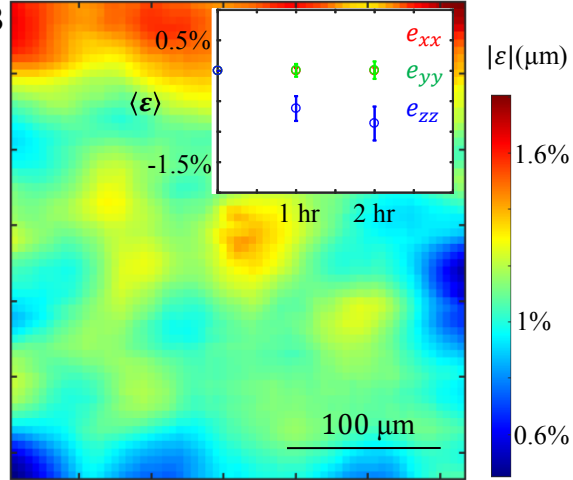

Supplement: S1 Fig — In order to estimate the noise due to systematic errors in the reflectance traction microscopy, we have taken 3 image stacks of a collagen gel sample without cells at 1 hour intervals. The image stacks have 1024 × 1024 × 60 pixels, where each voxel measures 0.36 × 0.36 × 0.5 μm. Using the first stack (0 hour) as a reference, we have computed the 3D deformation field and strain tensor on a spatial grid of 16 × 16 × 8 at 1 hour and 2 hour time points. (A) The magnitude of 3D deformation field at the horizontal middle plane of the imaging volume at 2 hours. Inset: average deformation along each direction. Red: Dx, Green: Dy, Blue: Dz. Overall, the magnitude of deformation due to systematic errors (such as mechanical drifts, temperature fluctuations) is less than 0.5 μm over 2 hours. (B) The magnitude of strain magnitude at the horizontal middle plane of the imaging volume at 2 hours. Inset: average strain tensor along each principle directions. Red: εxx, Green: εyy, Blue: εzz. Overall, the systematic errors of strain field is less than 1% over 2 hours. (PDF) [file pone.0156797.s001.pdf]

A

deformation (pixel)

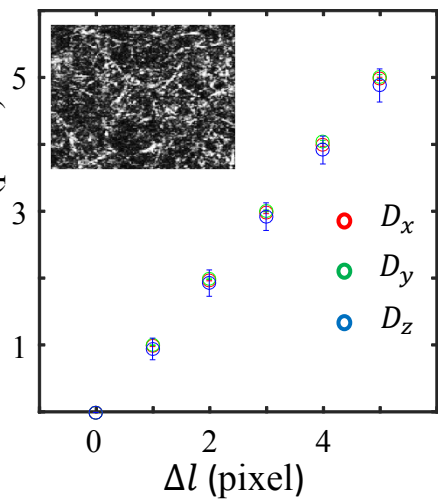

B

deformation (pixel)

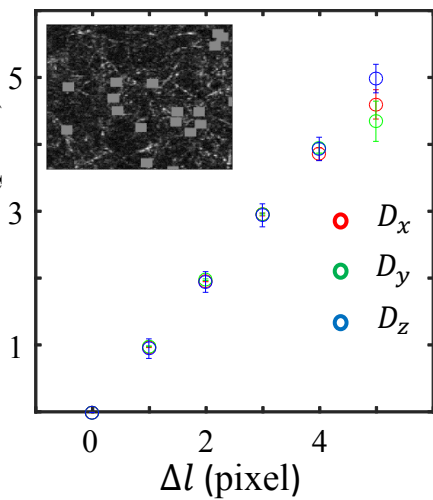

C

deformation (pixel)

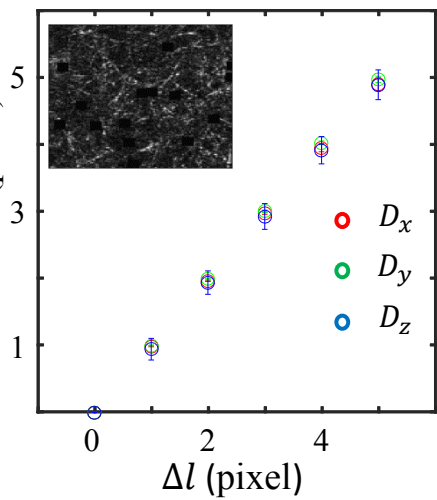

Supplement: S2 Fig — The ECM can be densified or degraded by cellular activities. To determine the effect of the ECM heterogeneity in the precision of reflectance traction microscopy, we have performed the test on simulated data sets. First of all, we have taken a section of confocal reflection images Iraw (128 × 128 × 64 pixels, available from https://github.com/bosunorst/Partial-Volume-Correlation/), and computationally translated Iraw by Δl pixels in all directions. We then applied PVC to compute the (theoretically uniform) deformation field [Dx, Dy, Dz] on a grid with 16 × 16 × 8 pixels spacing. Statistics of [Dx, Dy, Dz] then estimate the error of reflectance traction microscopy. (A) The means and standard deviations of [Dx, Dy, Dz] at varying Δl. The errors of the deformation fields are within 5%. Inset: a 2D section of the image stack Iraw. To simulate remodeled ECM, we have randomly chosen 110 cubes within Iraw. These cubes each have a size of 8 × 8 × 8 pixels. In total these cubes occupy ≈4% volume of Iraw. We fill the cubes by the value that corresponds to bright pixels (the upper 2% intensity in Iraw) to simulate images of densified ECM Iden, or we fill the cubes by the value that corresponds to dark pixels (the lower 2% intensity in Iraw) to simulate images of degraded ECM Ideg. Similar to (A), we have applied PVC on computationally translated Iden and Ideg, and the results are shown in (B-C). (B) The means and standard deviations of [Dx, Dy, Dz] at varying Δl for densified ECM. The errors of the deformation fields are within 5%, except when Δl > 4 pixels, where the errors are approaching 10%. Inset: a 2D section of Iden. (C) The means and standard deviations of [Dx, Dy, Dz] at varying Δl for degraded ECM. The errors of the deformation fields are within 5%. Inset: a 2D section of Ideg. (PDF) [file pone.0156797.s002.pdf]

A

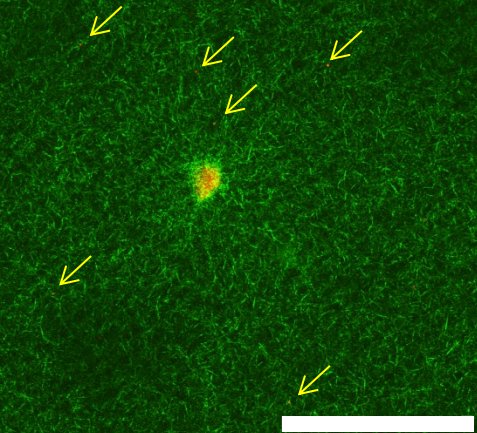

B

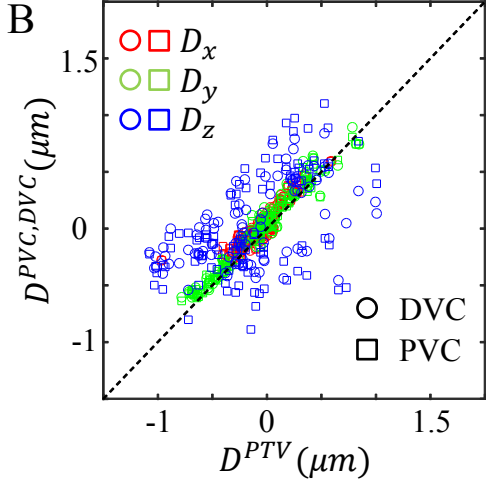

Supplement: S3 Fig — We have compared reflectance traction microscopy side by side with two other popular approaches: particle tracking velocimetry (PTV) and direct volume correlation (DVC, [33]). (A) We have seeded low density fluorescent tracing particles (red, 0.2 μm diameter, highlighted by the arrows) in collagen gels (green) containing MDA-MB-231 cells (red). The particle density is kept low so that (1) it does not bias the confocal reflection image; (2) it avoids ambiguities in trajectory reconstruction. Scale bar: 100 μm. (B) We have calculated deformation field induced by MDA-MB-231 cells on a spatial grid of 4 × 4 × 4 pixels, with each voxel measures 0.36 × 0.36 × 0.5 μm. We have applied both PVC and DVC methods on the reflectance images, and (linearly) interpolated the deformation field at particle locations to obtain DPVC and DDVC. On the other hand, we have calculated the particle displacements using their fluorescent images directly with PTV, resulting in DPTV. Results of ≈ 100 particles from three experiments are shown in B, where we compare DPVC, DDVC with respect to DPTV. The mean square deviations between PVC and PTV are 0.11 μm in x-direction, 0.08 μm in y-direction, 0.51 μm in z-direction. The mean square deviations between DVC and PTV are 0.11 μm in x-direction, 0.1 μm in y-direction, 0.47 μm in z-direction. Therefore PVC and DVC give very close results, and their deviation from PTV is comparable to the noise ground set by systematic errors (S1 Fig). (PDF) [file pone.0156797.s003.pdf]

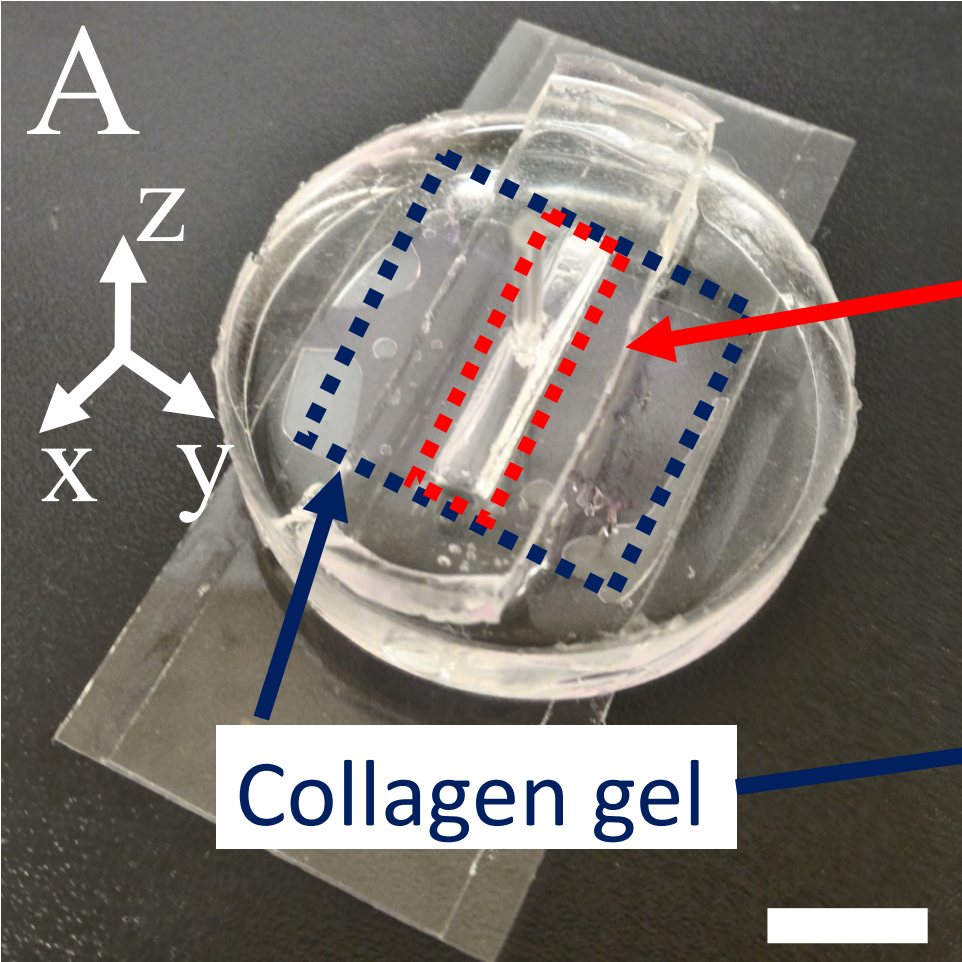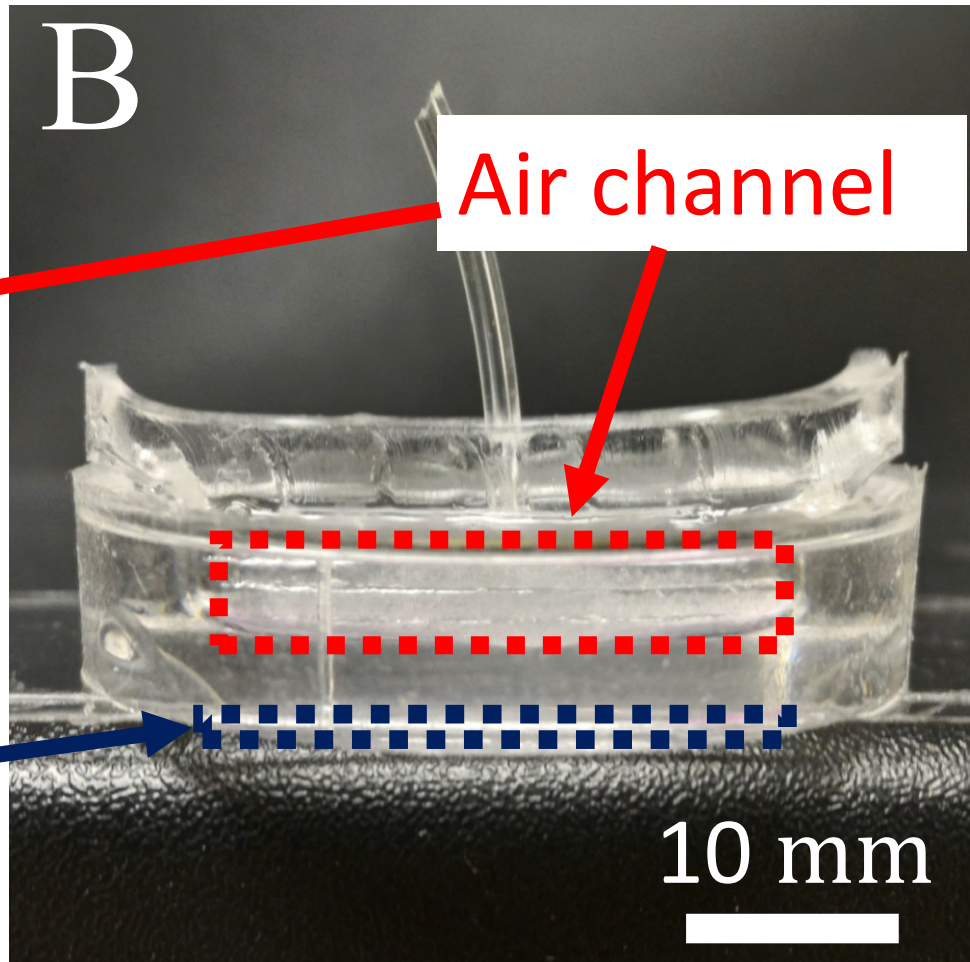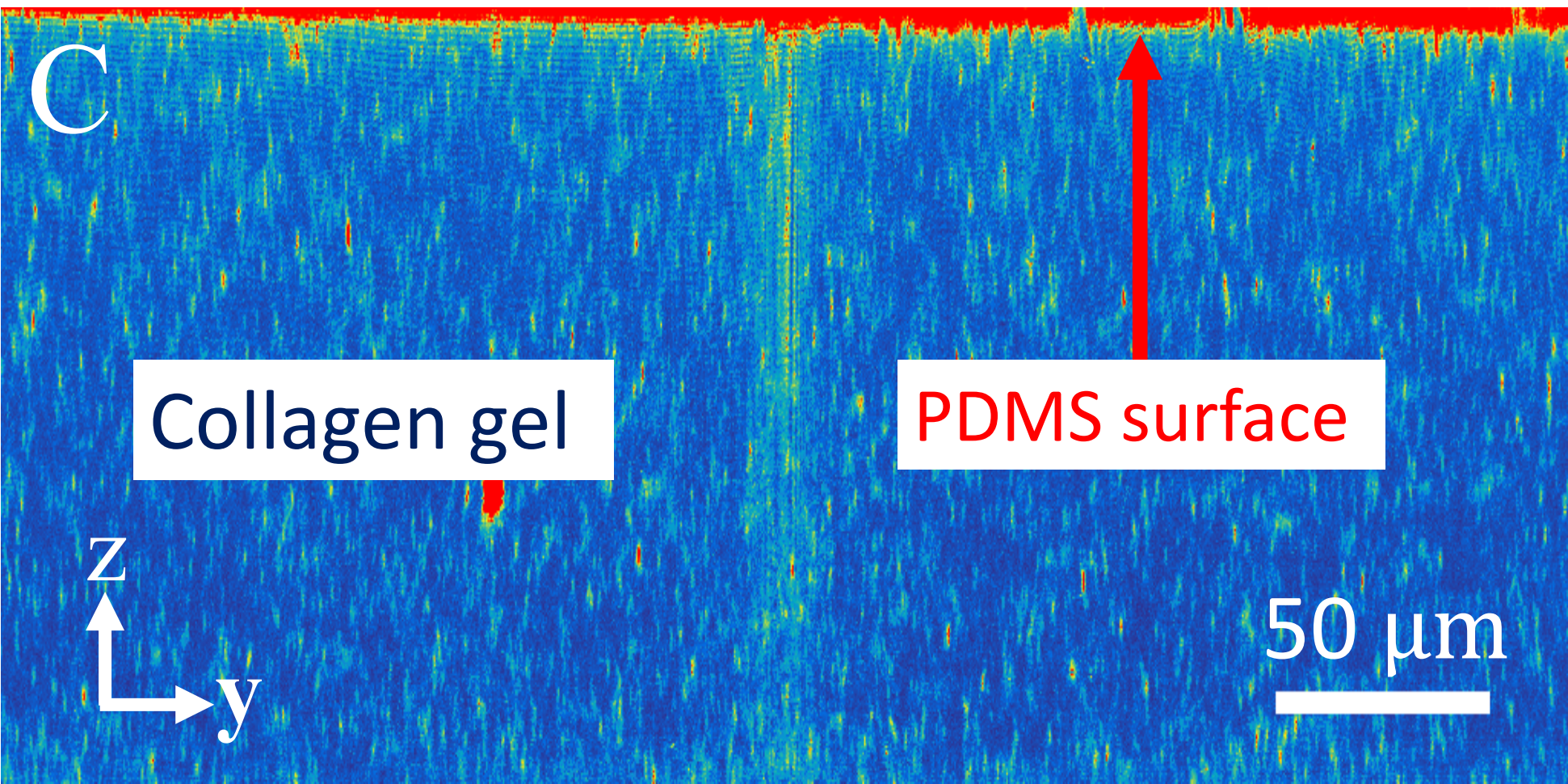

Supplement: S4 Fig — (A-B) The top and side view of the device. The red dashed lines indicate air channel and the blue lines show the region where collagen gel is placed. There is a thin PDMS membrane between air channel and collagen matrix. The membrane can be expanded or shrunk by controlling air volume through the tube. Scale bar: 10 mm. (C) Side view of the confocal reflection imaging of the sample. Green channel shows collagen fibers and red channel shows the PDMS membrane surface. This cross sectional image shows that the PDMS surface is slightly asymmetric due to the fabrication processes. (PDF) [file pone.0156797.s004.pdf]

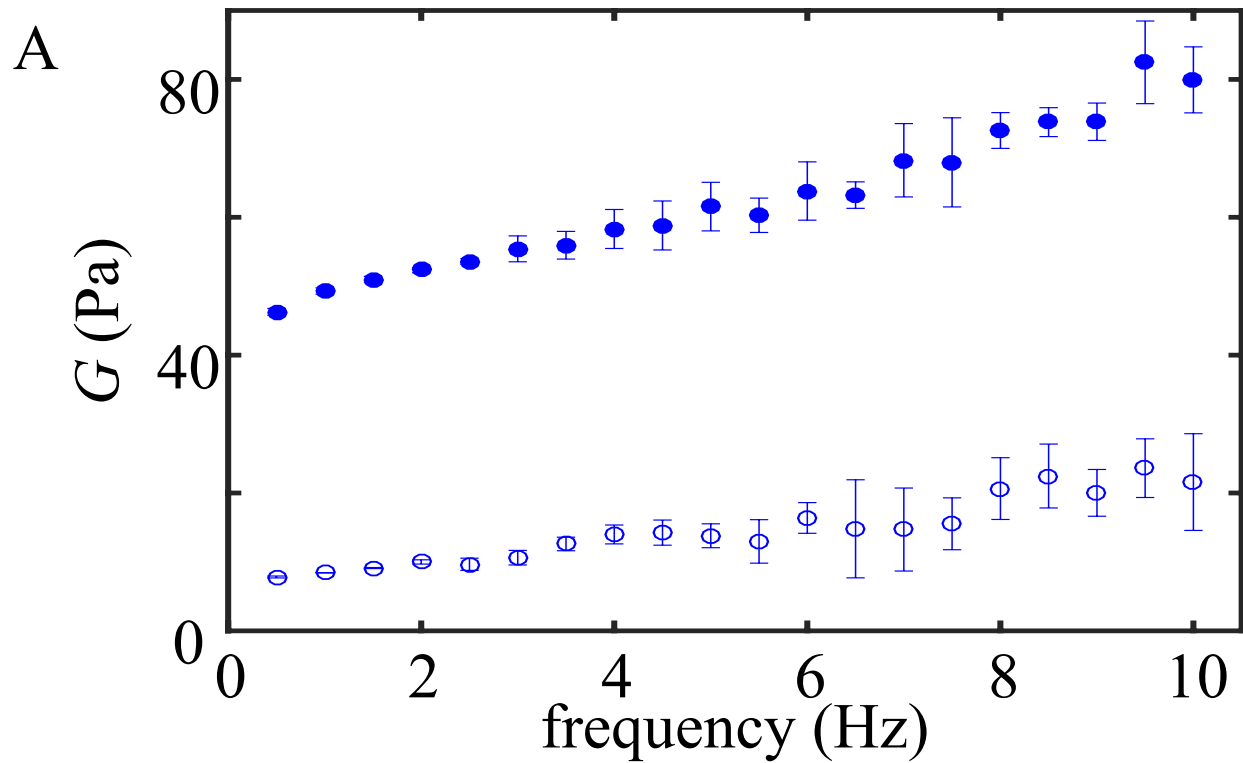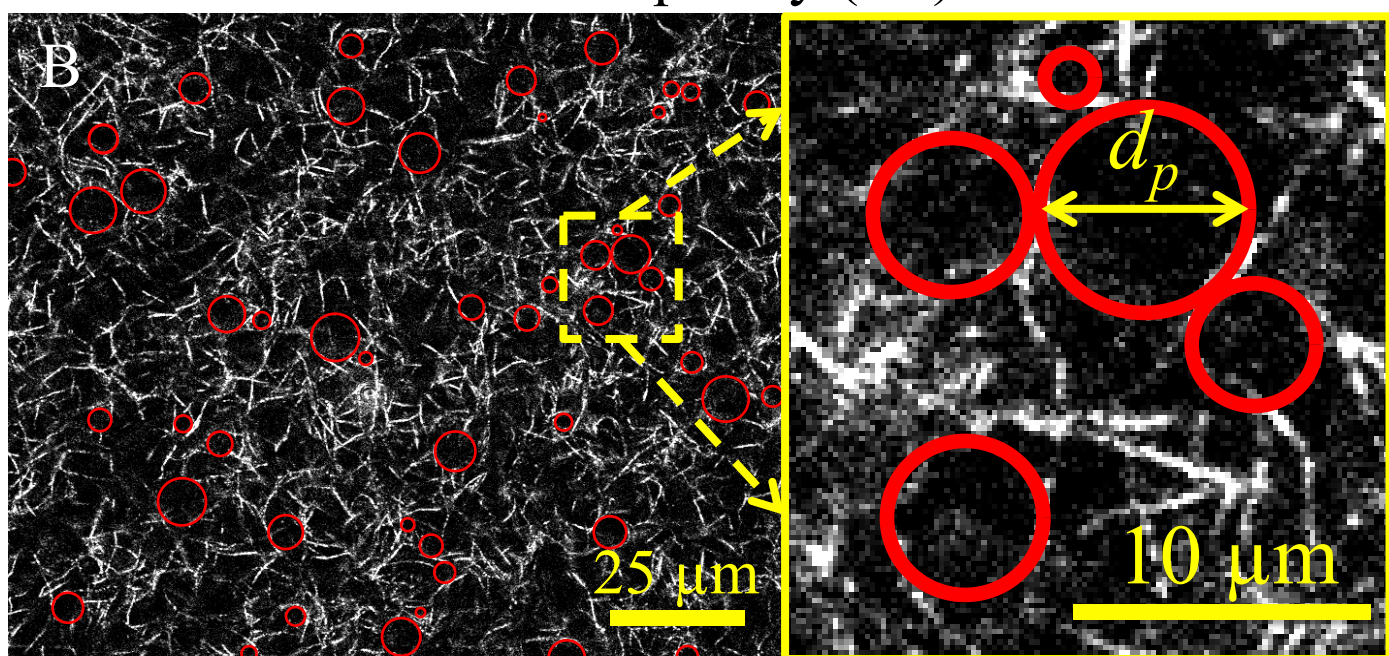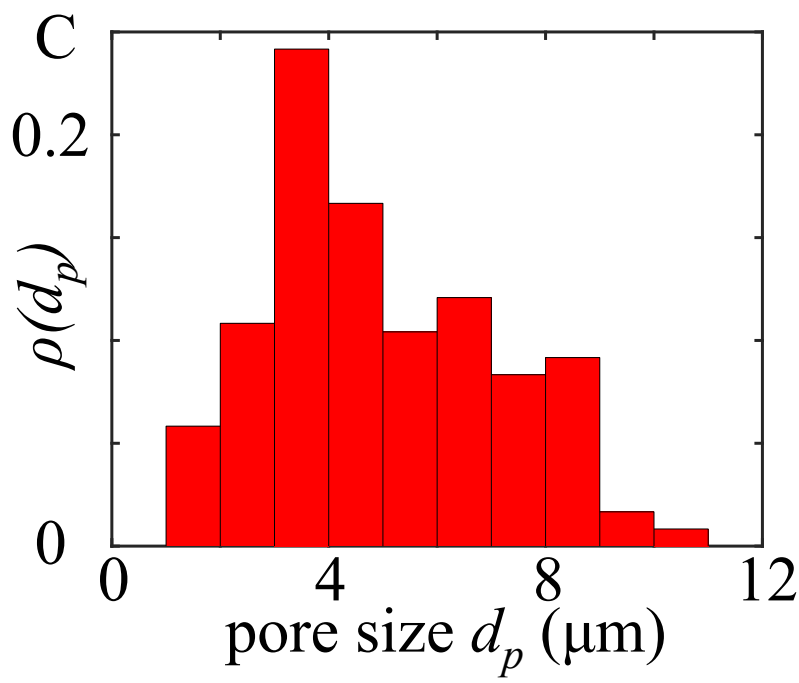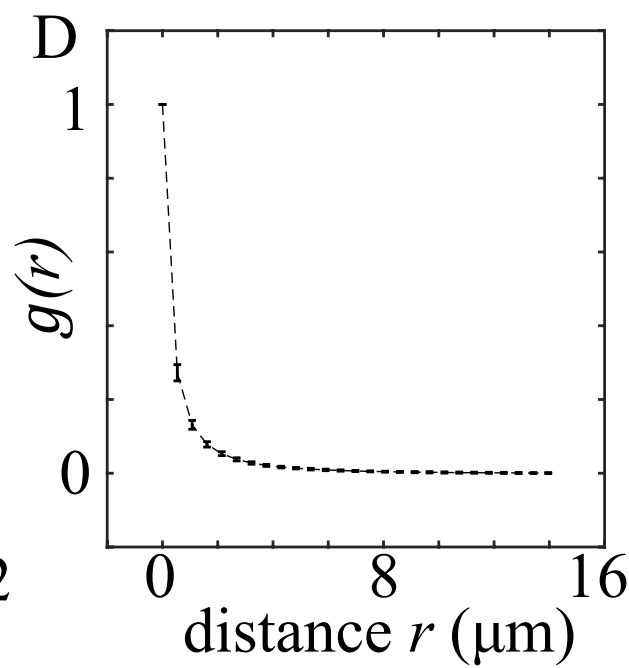

Supplement: S5 Fig — (A) The rheological modulus of the collagen gel as a function of frequency. Storage modulus G′ is plotted with solid circles and loss modulus G′′ is plotted with open circles. Measurements were taken using an AR 2000 rheometer with a 20 mm steel plate and Peltier plate for temperature control. Immediately after neutralization, collagen solution was injected between the plates, which had been preheated to 37°C. Silicone oil was allowed to polymerize for one hour before rheological measurements. Error bars show the standard deviation of five separate frequency sweeps on the same sample. (B) A sample confocal image slice with red circles representing pores in the collagen network. The pore size dp is defined as the diameter of the largest circle that can be drawn to fit inside the pore. 240 pores were randomly selected and the circles were drawn manually. (C) The pore size distribution of the collagen gel. Mean pore size is 4.95 μm. (D) Density fluctuation of the collagen gel as measured by 2-point intensity correlation g(r) [30]. Double exponential fit is given by g(r) = 0.8exp(−r/0.34μm) + 0.2exp(−r/1.9μm). (PDF) [file pone.0156797.s005.pdf]

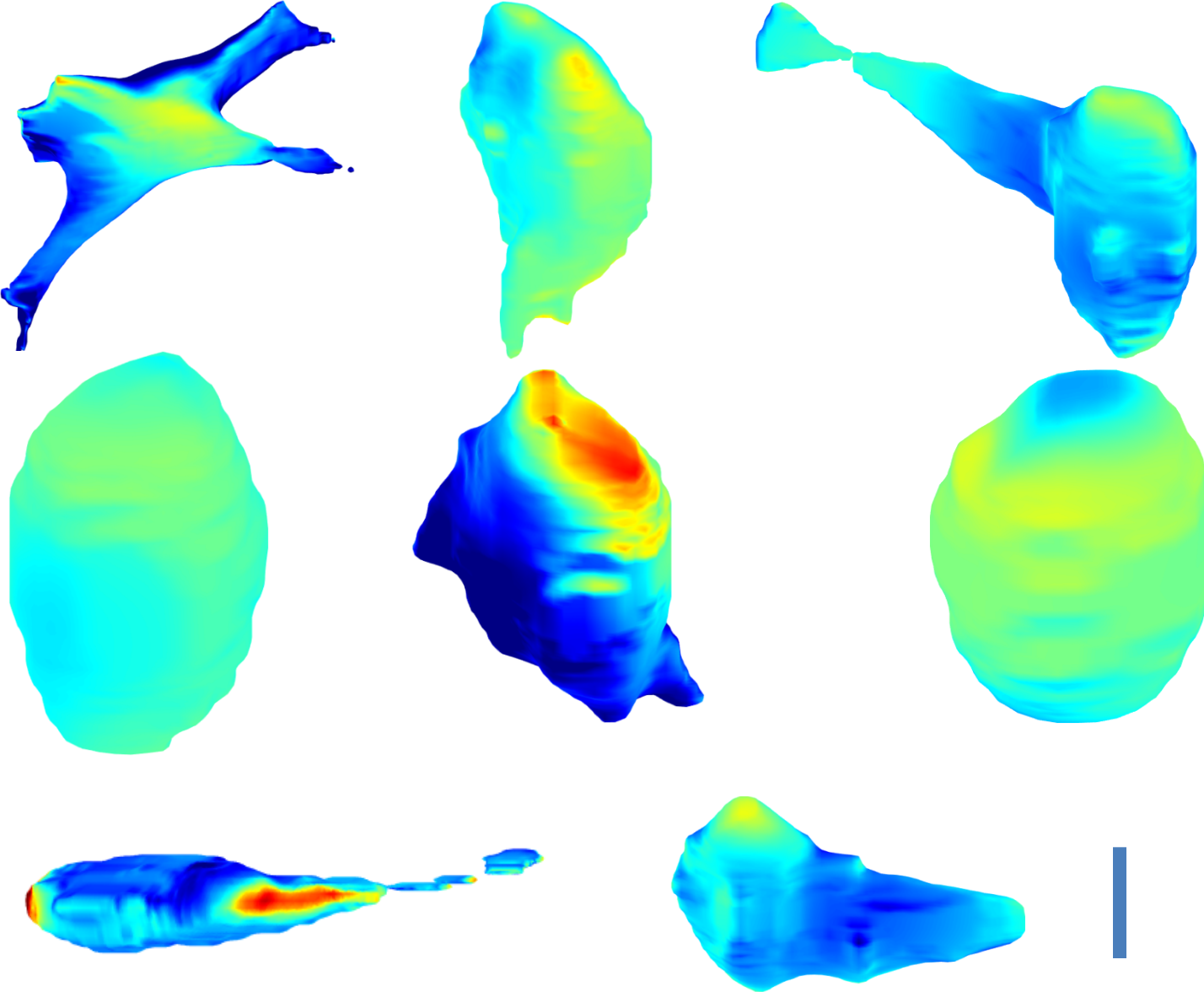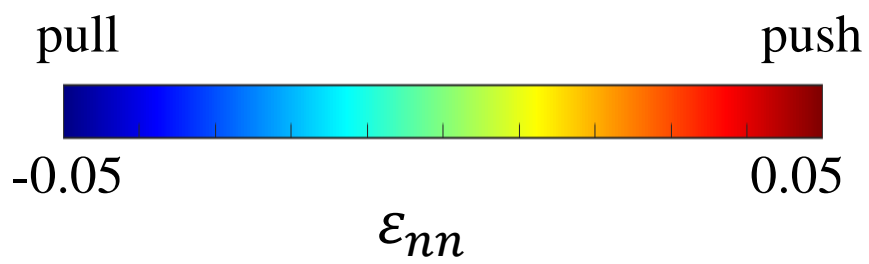

Supplement: S6 Fig — The traction field of cells can be qualitatively characterized by the normal projection of the strain field on to cell membrane. From the strain field εij, and the normal direction n of cell surface, we have calculated the normal projection εnn = n ⋅ ε ⋅ n. We have shown εnn of 8 cells on the same spatial and color scales (scale bar: 40 μm). Cells exert both pulling (εnn < 0) and pushing (εnn > 0) forces, corresponding to different cellular activities, such as newly formed protrusion and active contraction. (PDF) [file pone.0156797.s006.pdf]

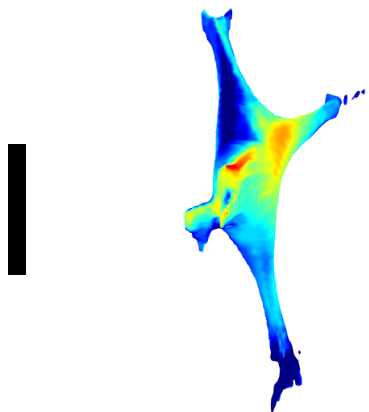

$C = 22.7$  nN

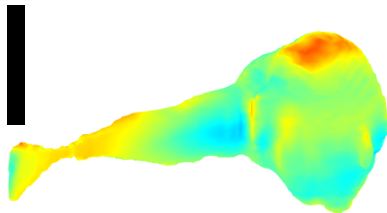

$C = 17.9$  nN

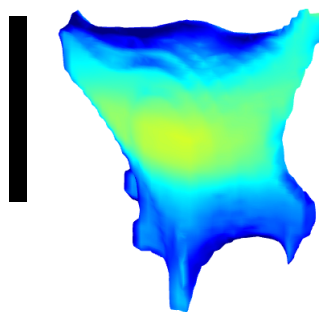

$C = 17.4$  nN

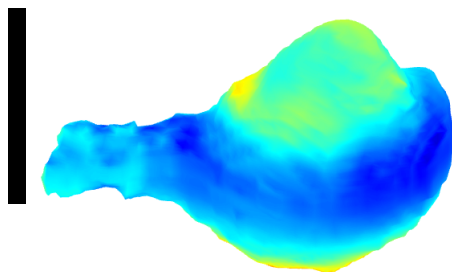

$C = 20.1$  nN

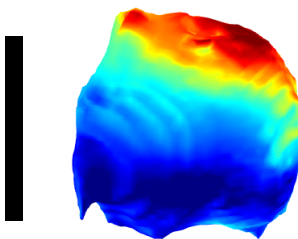

$C = 22.0$  nN

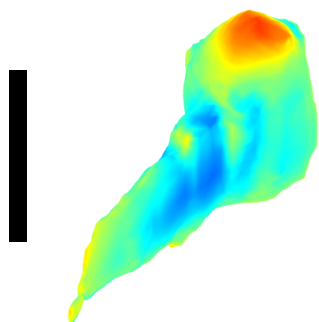

$C = 10.9$  nN

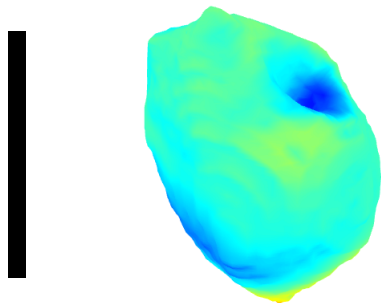

$C = 14.8$  nN

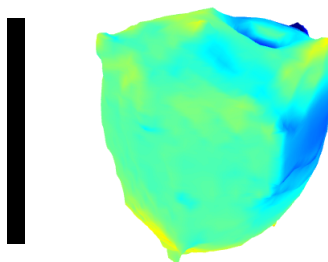

$C = 7.3$  nN

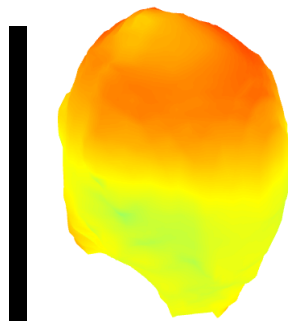

$C = 4.2$  nN

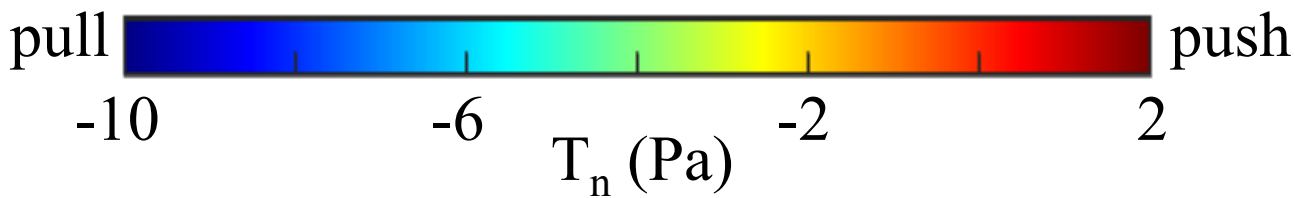

Supplement: S7 Fig — We calculate the cell traction assuming that the collagen network is an isotropic, homogeneous material. We make the linear elastic approximation because strain magnitudes are small (<5%). Using these approximations, the Cauchy stress tensor σ is given by σij = 2Gεij + λTrace(ε)δij where λ = 2Gν/(1 − 2ν), G is the shear modulus, ν is the Poission ratio, and δij is the Kronecker delta [46, 47]. We take G = 50 Pa and ν = 0.2 which is consistent with previous experimental results [47–50]. The traction T at the cell surface is calculated from the stress tensor using the Caucy relation T = n ⋅ σ where n are the directions normal to the cell surface [46, 47]. We generate a finite element mesh to represent the cell surface and then calculate the surface normal and traction for each discrete face. The normal component of traction Tn = T ⋅ n is shown for nine cells on the same spatial and color scale (scale bars: 40 μm). Negative normal traction represents pulling and positive normal traction represents pushing. We have found that the normal traction is generally the largest component of the total traction and that pulling tractions are generally stronger than pushing. Typical surface tractions in our experiments are ∼5–10 Pa which is consistent with previous results for cell traction in collagen gels [42], but much smaller than mean tractions ∼300 Pa reported for cells in synthetic PEG hydrogels [20]. In addition, we have calculated the contractility for each cell, which is defined as the total magnitude of force projected onto the direction of the center of mass of the cell C = ∑A T ⋅ Rcm, where A is the area of each face, Rcm is the direction of the center of mass, and the sum is over all faces. Typical contractility for our experiments is ∼15 nN which is slighlty smaller than the previously reported value of ∼45nN for cells in 3D collagen gels [42]. The discrepancy could arise from linear elastic approximation used here, compared with the constitutive equation and regula [file pone.0156797.s007.pdf]
